# Enhancement of Spontaneous Activity by HCN4 Overexpression in Mouse Embryonic Stem Cell-Derived Cardiomyocytes - A Possible Biological Pacemaker

**DOI:** 10.1371/journal.pone.0138193

**Published:** 2015-09-18

**Authors:** Yukihiro Saito, Kazufumi Nakamura, Masashi Yoshida, Hiroki Sugiyama, Tohru Ohe, Junko Kurokawa, Tetsushi Furukawa, Makoto Takano, Satoshi Nagase, Hiroshi Morita, Kengo F. Kusano, Hiroshi Ito

**Affiliations:** 1 Department of Cardiovascular Medicine, Okayama University Graduate School of Medicine, Dentistry, and Pharmaceutical Sciences, Okayama, Japan; 2 Sakakibara Heart Institute of Okayama, Okayama, Japan; 3 Department of Bio-informational Pharmacology, Medical Research Institute, Tokyo Medical and Dental University, Tokyo, Japan; 4 Department of Physiology, Kurume University School of Medicine, Kurume, Japan; 5 Department of Cardiovascular Therapeutics, Okayama University Graduate School of Medicine, Dentistry, and Pharmaceutical Sciences, Okayama, Japan; 6 Department of Cardiovascular Medicine, National Cerebral and Cardiovascular Center, Osaka, Japan; University of Milan, ITALY

## Abstract

**Background:**

Establishment of a biological pacemaker is expected to solve the persisting problems of a mechanical pacemaker including the problems of battery life and electromagnetic interference. Enhancement of the funny current (*I*_f_) flowing through hyperpolarization-activated cyclic nucleotide-gated (HCN) channels and attenuation of the inward rectifier K^+^ current (*I*_K1_) flowing through inward rectifier potassium (K_ir_) channels are essential for generation of a biological pacemaker. Therefore, we generated HCN4-overexpressing mouse embryonic stem cells (mESCs) and induced cardiomyocytes that originally show poor *I*_K1_ currents, and we investigated whether the HCN4-overexpressing mESC-derived cardiomyocytes (mESC-CMs) function as a biological pacemaker *in vitro*.

**Methods and Results:**

The rabbit *Hcn4* gene was transfected into mESCs, and stable clones were selected. mESC-CMs were generated via embryoid bodies and purified under serum/glucose-free and lactate-supplemented conditions. Approximately 90% of the purified cells were troponin I-positive by immunostaining. In mESC-CMs, expression level of the *Kcnj2* gene encoding K_ir_2.1, which is essential for generation of *I*_K1_ currents that are responsible for stabilizing the resting membrane potential, was lower than that in an adult mouse ventricle. HCN4-overexpressing mESC-CMs expressed about a 3-times higher level of the *Hcn4* gene than did non-overexpressing mESC-CMs. Expression of the *Cacna1h* gene, which encodes T-type calcium channel and generates diastolic depolarization in the sinoatrial node, was also confirmed. Additionally, genes required for impulse conduction including *Connexin40*, *Connexin43*, and *Connexin45* genes, which encode connexins forming gap junctions, and the *Scn5a* gene, which encodes sodium channels, are expressed in the cells. HCN4-overexpressing mESC-CMs showed significantly larger *I*_f_ currents and more rapid spontaneous beating than did non-overexpressing mESC-CMs. The beating rate of HCN4-overexpressing mESC-CMs responded to ivabradine, an *I*_f_ inhibitor, and to isoproterenol, a beta-adrenergic receptor agonist. Co-culture of human induced pluripotent stem cell-derived cardiomyocytes (hiPSC-CMs) with aggregates composed of mESC-CMs resulted in synchronized contraction of the cells. The beating rate of hiPSC-CMs co-cultured with aggregates of HCN4-overexpressing mESC-CMs was significantly higher than that of non-treated hiPSC-CMs and that of hiPSC-CMs co-cultured with aggregates of non-overexpressing mESC-CMs.

**Conclusions:**

We generated HCN4-overexpresssing mESC-CMs expressing genes required for impulse conduction, showing rapid spontaneous beating, responding to an *I*_f_ inhibitor and beta-adrenergic receptor agonist, and having pacing ability in an *in vitr*o co-culture system with other excitable cells. The results indicated that these cells could be applied to a biological pacemaker.

## Introduction

The establishment of a biological pacemaker is expected to solve the persisting problems of a mechanical pacemaker including the problems of battery life, lead breaks, infection, electromagnetic interference, appearance, and heart rate response during exercise.

Cardiac pacemaker activity originates in the sinus node. Spontaneous diastolic depolarization in phase 4 of the action potential is initiated in the sinus node, and the electrical impulse is conducted through the atria to the atrioventricular node. The sinus node can generate impulses faster than those generated in other areas. Different kinds of ionic currents are involved in the formation of spontaneous diastolic depolarization of the sinus node [1–6].

Among them, the funny current (*I*_f_) flowing through hyperpolarization-activated cyclic nucleotide-gated potassium (HCN) channel is robustly present in the sinus node [7]. Since the HCN4 isoform is mainly expressed in the sinus node [8, 9] and contains the cAMP-binding domain, adrenergic stimulation followed by intracellular cAMP accumulation increases the heart rate through augmentation of *I*_f_ currents [7, 10]. We previously reported a mutation of *HCN4* found in a patient suffering from sick sinus syndrome (SSS) [11]. Other groups also reported an association between SSS and mutation of *HCN4* [12, 13]. Therefore, HCN4 is thought to be the key pacemaking ion channel [8].

In contrast, the inward rectifier K^+^ current (*I*_K1_), which maintains resting membrane potential in working myocytes and antagonizes spontaneous activity, is negligibly small in sinus node cells [14, 15]. Interestingly, genetic suppression of *I*_K1_ can give rise to pacemaker activity in ventricular myocytes [16]. Despite the presence of *I*_K1_, Purkinje fibers show *I*_f_-dependent pacemaking, but the pacemaking is slow and not robust. Thus, one of the most important factors responsible for the rapid and robust pacemaking of the sinus node is the absence of *I*_K1_ [1, 17].

That is, enhancement of *I*_f_ flowing through HCN channels and attenuation of *I*_K1_ flowing through K_ir_ channels are required for pacemaker cells to undergo spontaneous diastolic depolarization. Therefore, we generated HCN4-overexpressing mouse embryonic stem cells (mESCs) and induced cardiomyocytes that originally show weak *I*_K1_ currents to achieve two prerequisites for forming spontaneous diastolic depolarization, large *I*_f_ currents and small *I*_K1_ currents, and investigated whether the HCN4-overexpressing mESC-derived cardiomyocytes (mESC-CMs) function as a biological pacemaker *in vitro*.

## Materials and Methods

### Plasmid construction

Vertebrate HCN4 proteins are highly conserved [11] and we used the rabbit *Hcn4* in mESCs due to the traceability. CAG promoter-IRES-*EGFP* construct in pCAGIG (Addgene #11159) and kanamycin/neomycin resistance gene (*Kan* ^*R*^ */Neo* ^*R*^) in pIRES2-*AcGFP1* vector (Clontech) were amplified via the polymerase chain reaction (PCR) method using PrimeSTAR GXL DNA polymerase (TaKaRa), purified using a QIAquick PCR Purification Kit (Qiagen), and ligated using an In-fusion HD enzyme (Clontech), i.e., pCAGIG- Kan^R^/Neo^R^. Rabbit *Hcn4* cDNA in the pCI vector (previously reported and kindly provided by Dr Takano) [9, 11] was amplified via the PCR method and ligated with pCAGIG-*Kan* ^*R*^ */Neo* ^*R*^, i.e., pCAGIG-rabbit *Hcn4*-*Kan* ^*R*^ */Neo* ^*R*^. PCR primers are shown in Table 1.

**Table 1:** Primer sets used for subcloning, RT-PCR and quantitative PCR.

Genes		Sequences	Annealing temperature	Cycles
**Subcloning**				
CAG-IRES-*EGFP*	forward	CGGTTCCTCTAGTTATTAATAGTAATCAATTACG	71	25
	reverse	ATATTTGAACTGCAGGTCGAGGGATCT		
*Kan* ^*R*^ */Neo* ^*R*^	forward	CTGCAGTTCAAATATGTATCCGCTCA	71	25
	reverse	ATAACTAGAGGAACCGTAAAAAGGCC		
rabbit *Hcn4*	forward	CGAATTCACCATGGACAAGCTGCCGCCGTC	71	25
	reverse	CCTCGAGTCACAGGTTGGACGGCAGTTTG		
**RT-PCR**				
*Gapdh*	forward	CATGGCCTTCCGTGTTCCTA	58	25
	reverse	TGCCTGCTTCACCACCTTCT		
*Oct4*	forward	AGATCACTCACATCGCCAAT	57	25
	reverse	AAGGTGTCCTGTAGCCTCAT		
*Nanog*	forward	GCAAGAACTCTCCTCCAT	57	25
	reverse	ATACTCCACTGGTGCTGA		
*Nkx2*.*5*	forward	CGACGGAAGCCACGCGTGCT	57	35
	reverse	CCGCTGTCGCTTGCACTTG		
*Tnnt2*	forward	CAGGAAAAGTTCAAGCAGCA	62	35
	reverse	GCTCCCACTATCCAAACAGG		
*Scn5a*	forward	CTTGGCCAAGATCAACCTGCTCT	57	35
	reverse	CGGACAGGGCCAAATACTCAATG		
*Cacna1h*	forward	GCTGTTTGGGAGGCTAGAAT	57	35
	reverse	CGAAGGTGACGAAGTAGACG		
rabbit *Hcn4*	forward	GTACTCCTACGCGCTCTTCA	57	30
	reverse	GCTCTCCTCGTCGAACATCT		
mouse *Hcn4*	forward	GGATTATCCACCCCTACAG	60	30
	reverse	GTCTCGCCAAGTCAATGAGGAAGAAT		
*Gja5*	forward	CCACGGAGAAGAATGTCTTCA	55	35
	reverse	TGCTGCTGGCCTTACTAAGG		
*Gja1*	forward	TGGGGGAAAGGCGTGAG	55	35
	reverse	CTGCTGGCTCTGCTGGAAGGT		
*Gjc1*	forward	ATCATCCTGGTTGCAACTCC	57	35
	reverse	CTCTTCATGGTCCTCTTCCG		
**quantitative PCR**				
*β-Actin*	forward	GGAGGGGGTTGAGGTGTT	61	40
	reverse	GTGTGCACTTTTATTGGTCTCAA		
*Kcnj2*	forward	GCTGGTCAAAAGAACCCCAAGG	61	40
	reverse	TTCCCTCCGAAGAGACGATGCTG		
*Gapdh*	forward	CATGGCCTTCCGTGTTCCTA	55	40
	reverse	TGCCTGCTTCACCACCTTCT		
total *Hcn4*	forward	CCCATGCTGCAGGACTTC	55	40
	reverse	GCTTCCCCCAGGAGTTATTC		

### Maintenance of mESCs

Mouse ESCs (cell line CGR8; ECACC) were cultivated on 0.1% gelatin-coated plates in high-glucose Dulbecco’s Modified Eagle’s medium (DMEM; GIBCO) supplemented with 20% fetal bovine serum (Sigma), 50 μM ß-mercaptoethanol (2-ME), MEM nonessential amino acids solution (NEAA, GIBCO), 1000 units/mL leukemia inhibitory factor (LIF; WAKO), and 100 μg/mL kanamycin (Sigma) in a humidified atmosphere containing 5% CO_2_.

### Nucleofection

CGR8 cells were harvested using 0.05% trypsin/EDTA. Two μg of a non-linearized vector was used for nucleofection (Amaxa Nucleofector Ⅱ; A-023, which is optimized for a nucleofection program for mouse ESCs) and HCN4-overexpressing ESCs were selected using a medium containing 400 μg/ml G418 (Roche Applied Science) for 7 days. Three stable clones that were resistant to G418 and were EGFP-positive were selected and expanded.

### Electrophysiology

The funny current (*I*_f_) was recorded at room temperature by using the perforated patch-clamp technique. Cells were superfused with a bath solution containing (in mM): 132 NaCl, 4.8 KCl, 2.0 CaCl_2_, 1.2 MgCl_2_, 1.0 BaCl_2_, 2.0 MnCl_2_, 5.0 D-glucose, and 10 Hepes; pH 7.4. Pipettes (2–4 MΩ resistances) were filled with a pipette solution containing (in mM): 110 K-aspartate, 5.0 K_2_-ATP, 11 EGTA, 1.0 CaCl_2_, 1 MgCl_2_, and 5 Hepes; pH 7.2. Then 0.3 mg/mL Amphotericin B was added to the pipette solution to achieve patch perforation (10–20 MΩ; series resistance). The *I*_f_ current was activated by a standard activation protocol. *I*_f_ currents through activated HCN4 channels were obtained during hyperpolarizing test pulses of 5 seconds between -45 and -125 mV in 20 mV increments from a holding potential of -35 mV.

Action potentials (APs) were also measured with the perforated patch‐clamp technique. mESC-CMs were dissociated using 0.25% Trypsin/0.02% EDTA for 5minutes and resuspended in high-glucose DMEM supplemented with 20% FBS, 50 μM 2-ME, and NEAA. Then 1 x 10^5^/cm^2^ cells were replated on Matrigel-coated cover glasses and incubated for 48 hours. AP recordings were performed on monolayer cardiomyocytes. APs were measured respectively by the perforated patch‐clamp technique using an Axopatch 200B amplifier (Molecular Devices). Data acquisition of APs were performed with pClamp10.2/Clampfit (Axon Instruments). APs were measured using a modified Tyrode’s solution containing (in mM): 140 NaCl, 5.4 KCl, 1.8 CaCl2, 1.0 MgCl2, 5.5 glucose, and 5.0 HEPES; pH 7.4 (NaOH). The pipette solution contained (in mM) 110 DL-aspartic acid, 30 KCl, 1 CaCl_2_, 5 ATP-Mg, 5 Creatine P-Na, 5 HEPES, and 10 EGTA; pH 7.25 (KOH). To achieve patch perforation (series resistance: 10–20 MΩ), amphotericin B (0.3 mg/mL) (Nacalai Tesque, Inc., Kyoto) was added to the pipette solution. Temperature was maintained at 35–36°C by a TC-344B dual channel heating system (Warner Instruments).

### Cardiac differentiation of mESCs

Emryoid bodies (EBs) were formed by cultivating 500 mESCs with 0.5 mmol/L 2-O-alpha-D-glucopyranosyl-L-ascorbic acid (AA-2G; Hayashibara Biochemical Labs) and without LIF in a hanging drop for 5 days (culture day 0 to day 5)[18]. On day 5, EBs were collected and plated on a 0.1% gelatin-coated dish with a medium containing 0.25 mM AA-2G and 10 μM IWR-1-endo (WAKO). On day 7, the medium was exchanged a medium consisting of modified Eagle’s medium (MEM; GIBCO), Insulin-Transferrin-Selenium-A supplement (100×; ITS-A supplement; GIBCO), and 100 μg/mL kanamycin. The medium was changed every other day after plating on the dishes.

On day 14, the medium was changed to no glucose DMEM (Gibco) with 4 mM L-sodium lactate (Sigma-Aldrich)[19]. Until day 21, the medium was changed every other day. On day 21, EBs were treated with 0.25% Trypsin/EDTA (invtrogen) at 37°C for 5 minutes and dissociated. Dissociated cells were resuspended in 3 ml of medium and loaded onto a discontinuous Percoll (GE Healthcare) gradient, containing 20 mM HEPES and 150 mM NaCl. The gradient consisted of 3 ml of a 40.5% Percoll layer over 3 ml of a 58.5% Percoll layer. After centrifugation at 1,500 xg for 30 minutes, cell layers were apparent. Cells at a 58.5% layer were collected [20]. The purified cells were resupended in high-glucose DMEM supplemented with 20% FBS, 1% NEAA, and 100 μM 2-ME.

### Generation of human induced pluripotent stem cells (hiPSCs)

To investigate pacing ability of HCN4-overexpressing mESC-CMs, we used a co-culture system with human induced pluripotent stem cell-derived cardiomyocytes (hiPSC-CMs).

Human dermal fibroblasts (HDFs) were obtained from abdominal skin of 41-year-old healthy Japanese male, the corresponding author of this article, by punch biopsy. The skin biopsy sample was dissected into 10 evenly sized pieces. The dissected pieces were plated in 6-well plates containing DMEM supplemented with 10% FBS and 0.1 mg/mL kanamycin and placed in a 37°C incubator. The medium was changed every 3 days. After reaching confluence, the cells were dissociated with trypsin/EDTA and passaged.

hiPSCs were generated from HDFs as previously reported [21]. HDFs were transfected with human *OCT4*, *SOX2*, *KLF4* and *c-MYC* cDNA (plasmid #17217, #17218, #17219, and #17220, Addgene) twice using retroviral vectors produced by Platinum Retrovirus Expression System, Pantropic (VPK-302, Cell Biolabs, Inc). Two days after retrovirus transfection, 1 x 10^5^ HDFs dissociated with 0.05% trypsin/0.02% EDTA were replated on 10 cm dishes with an SNL feeder layer (07032801, ECACC) and maintained in DMEM/F12 (GIBCO) supplemented with 20% knockout serum replacement (GIBCO), 100 μmol/L 2-ME, NEAA, 10 ng/mL basic fibroblast growth factor (bFGF; WAKO), 0.05 mmol/L AA-2G and 100 μg/mL kanamycin (Sigma). Twenty-five days after replating, hiPSC colonies were selected. The hiPSC colonies were expanded on the SNL feeder layer. For feeder-free culture, hiPSCs were maintained on human embryonic stem cell-qualified Matrigel (Corning) in TeSRE8 medium (Stemcell Techonologies).

### Cardiac Differentiation of hiPSCs

hiPSCs were dissociated into single cells with StemPro Accutase Cell Dissociation Reagent (GIBCO) and seeded on Matrigel-coated dishes at 1 x 10^5^/cm^2^ in TeSRE8 medium supplemented with 10 μM Y27632 (WAKO). Five days after plating, at day 0, cells were treated with 6 μM CHIR99021 (Cayman) and 50 μM AA-2G in RPMI medium (GIBCO) supplemented with B27-insulin supplement (GIBCO) and kanamycin. After 24 hours, at day 1, the medium was changed to RPMI supplemented with B27-insulin and 50 μM AA-2G. On day 3, 5 μM IWR-1-endo (WAKO) was added and was removed during the medium change on day 5. Cells were maintained in RPMI supplemented with B27 supplement (GIBCO) starting from day 7, with the medium was changed every 2 or 3 days. On day 28, cardiomyocyte purification was performed by the Percoll gradient procedure mentioned above.

### Reverse Transcription-PCR (RT-PCR) and quantitative PCR

Total RNA from mESC-CMs was extracted using a Trizol Plus RNA Purificarion Kit (Invitrogen). Complementary DNA was synthesized from 1 μg of total RNA using a QunatiTect Reverse Transcription Kit (QIAGEN) as prescribed in the manual and subjected to PCR amplification.

Taq DNA polymerase (Roche Applied Science) was used for RT-PCR and PCR products were subjected to electrophoresis in 2% agarose gels and stained with ethidium bromide. RT-PCR experiments were performed twice in each of the 3 cell lines. SYBR Green PCR Master Mix and Applied Biosystems 7300 Real-Time PCR Systems (Applied Biosystems) were used for quantitative PCR (q-PCR). The q-PCR data were processed with a standard curve method. PCR primers are shown in Table 1. The q-PCR experiments were performed in triplicate in each 3-cell line.

### Immunocytofluorescence

mESCs, mESC-CMs and hiPSC-CMs were plated on matrigel-coated cover glasses and were fixed in 4% paraformaldehyde. Cells were stained with primary antibodies against OCT4 (1:50 dilution, Santa Cruz), EGFP (1:200 dilution, Frontier Institute), α-Actinin (1:800 dilution, Sigma EA-53), Troponin I (1:50 dilution, Santa Cruz), MLC-2v (1:50 dilution, ProteinTech Group) and MLC-2a (1:50 dilution, Synaptic Systems). Secondary antibodies were FITC-conjugated rabbit anti-goat IgG antibody, TRITC-conjugated rabbit anti-mouse IgG antibody, TRITC-conjugated swine anti-rabbit IgG (1:20 dilution, DAKO), and Alexa Fluor 488 goat anti-mouse IgG (1:200 dilution, Molecular Probes). Nucleus staining was performed with Hoechst 33342 (1:2500 dilution, Molecular Probes). F-actin staining was performed with Rhodamine Phalloidin (1:200 dilution, Molecular Probes).

### Counting beating rates of mESC-CMs

The purified mESC-CMs were plated on a laminin (Sigma Aldrich)-coated 96-well plate at 4 x 10^4^ cells/well in high-glucose DMEM supplemented with 20% FBS, 1% NEAA, and 100 μM 2-ME and were incubated for 4 days.

We counted spontaneous beating frequencies and examined responses to ivabradine and isoproterenol.

### Counting beating rates of hiPSC-CMs in a co-culture system with mESC-CMs

Aggregates containing 1 x 10^4^ mESC-CMs were made by using the hanging drop method for 3 days. The purified hiPSC-CMs were plated on a Matrigel-coated 24-well plate at 2 x 10^4^ cells/well and maintained in RPMI supplemented with B27. Three days after plating of hiPSC-CMs, the aggregate of mESC-CMs was plated on the well layered with hiPSC-CMs. After 7 days, synchronized beating rates of hiPSC-CMs away from aggregates were counted.

### Statistical analysis

All data are expressed as means ± SD. Statistical analysis was performed by student’s *t* test for unpaired data or one-way ANOVA with comparison of different groups by Dunnett’s *post hoc* test. Values of P < 0.05 were considered to be significant.

## Results

### Generation of mESC lines stably overexpressing rabbit *Hcn4*

**Fig 1:**
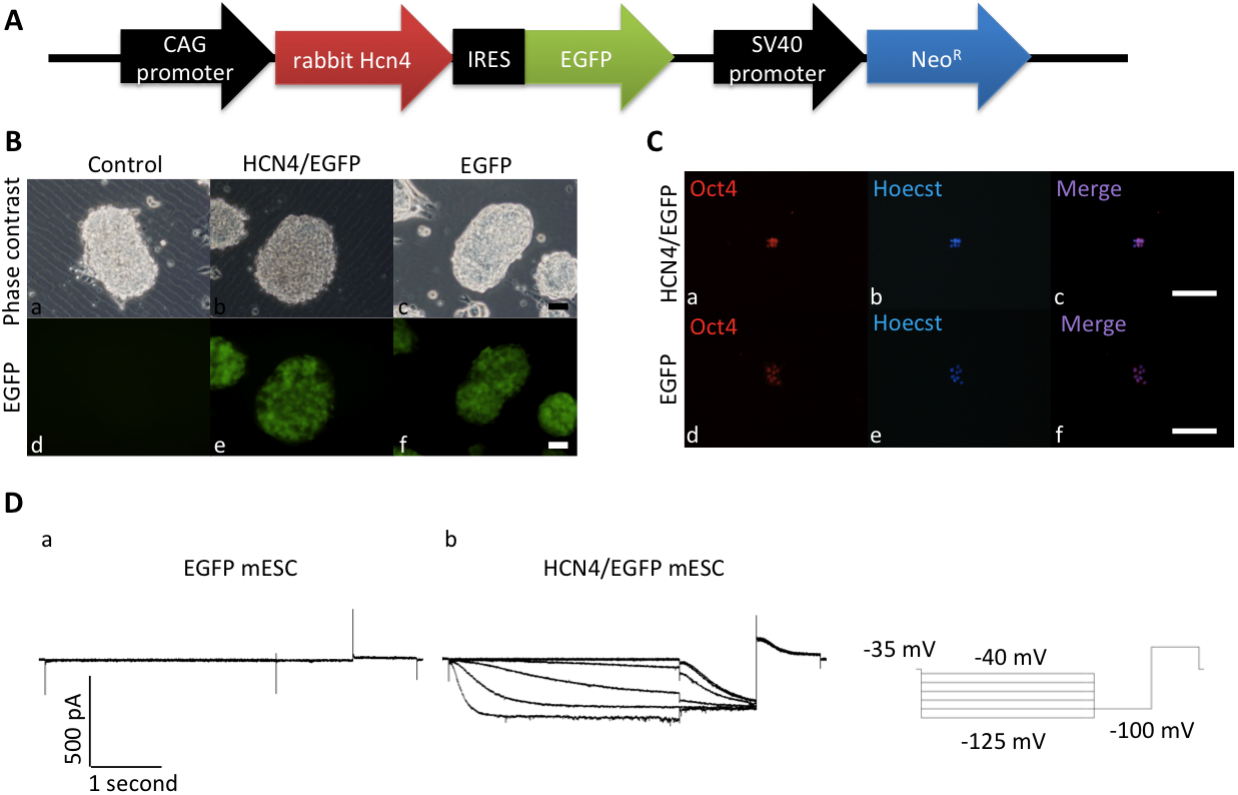
Generation of mESC lines stably overexpressing rabbit *Hcn4*. A. A transfection construct bearing the rabbit *Hcn4*-IRES-*EGFP* cassette. B. Representative living mESCs observed by phase contrast microscopy (a to c) and fluorescence microscopy (d to f). *Hcn4/EGFP* or *EGFP*-stably-transfected mESCs (HCN4/EGFP mESC-2 and EGFP mESC-1 cell lines) were positive for EGFP (green) fluorescence (e and f). C. Immunofluorescence staining of a pluripotency marker, OCT4, in *Hcn4/EGFP*-stably-transfected mESCs (a to c) and *EGFP*-stably-transfected mESCs (d to f). OCT4 was expressed in both mESC lines. Bar = 50 μm. D. Measurement of *I*_f_ currents in *EGFP*-stably-transfected mESCs (a) and *Hcn4/EGFP*-stably-transfected mESCs (b). Activation of the *I*_f_ current was demonstrated in *Hcn4/EGFP*-stably-transfected mESCs. *I*_f_ currents through activated HCN channels were obtained during hyperpolarizing test pulses of 5 seconds between -45 and -125 mV in 20 mV increments from a holding potential of -35 mV.

A CAG promoter-rabbit *Hcn4*-IRES-*EGFP*/SV40 promoter-neomycin resistance gene plasmid vector (HCN4/EGFP vector) (Fig 1A) or CAG promoter-IRES-*EGFP*/SV40 promoter-neomycin resistance gene plasmid vector (EGFP vector) was transfected in mESCs, and colonies of cells that were resistant to G418 and were EGFP-positive were selected (Fig 1B and S1 Fig). We generated 3 mESC lines in each group (HCN4/EGFP or EGFP-stably-transfected mESCs group). All 3 selected HCN4/EGFP-stably-transfected mESC lines expressed rabbit *Hcn4* (S2 Fig).

**Fig 2:**
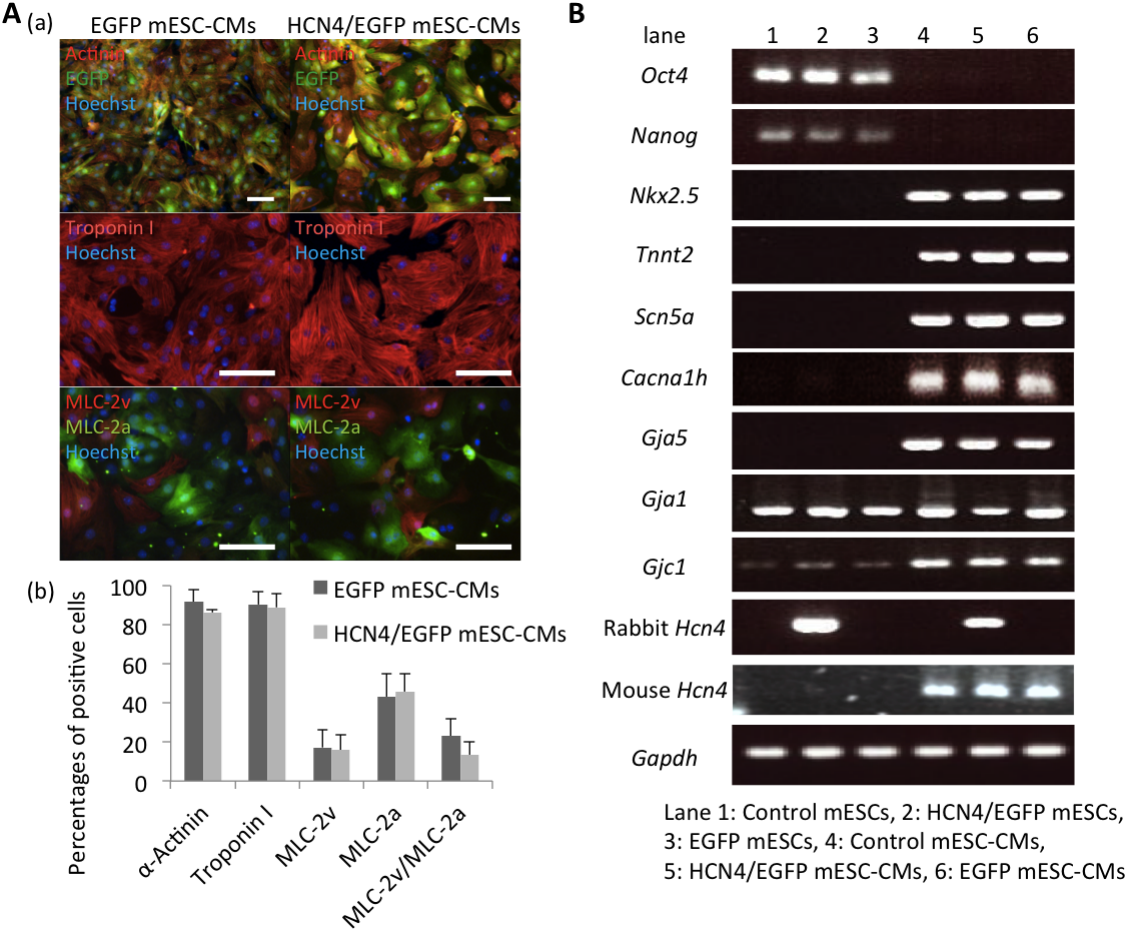
Establishment of purified HCN4-overexpressing mESC-CMs. A. (a) Representative immunofluorescence staining in *EGFP*-(left) or *Hcn4/EGFP-*(right) overexpressing mESC-CMs. α-actinin (red), EGFP (green) and Hoechst nuclear staining (blue) in the upper panel, troponin I (red) and Hoechst nuclear staining (blue) in the middle panel and myosin light chain (MLC)-2v (red), MLC-2a (green) and Hoechst nuclear staining (blue) in the bottom panel. (b) Percentages of immunofluorescence-positive cells for α-actinin (n = 7 in each group), EGFP, troponin I (n = 7 in each group), and MLC-2v and MLC-2a (n = 8 in EGFP mESC-CMs, n = 6 in HCN4/EGFP mESC-CMs). Data are expressed as mean ± SD. Bar = 50 μm. B. RT-PCR showed increases in mRNA expression for cardiac markers *Nkx 2*.*5*, *Tnnt2*, *connexin*, *Scn5a*, *Cacna1h*, and mouse endogenous *Hcn4* in mESC-CMs with or without HCN4 overexpression (lane 4 to 6). Rabbit exogenous *Hcn4* was expressed only in rabbit HCN4-transfected mESCs (lane 2) and mESC-CMs (lane 5).

As shown by immunostaining, RT-PCR and q-PCR, expression levels of *Oct4* (Figs 1C and 2B and S2 and S3 Figs) and *Nanog* (Fig 2B and S2 and S3C Figs), which are essential for maintaining the self-renewing and undifferentiated state in mESCs, were not influenced by HCN4 overexpression in any of the 3 cell lines.

*I*_f_ currents through activated HCN channels could be obtained during a hyperpolarizing test in HCN4-overexpressing mESCs (in HCN4/EGFP mESCs-2 cell line, n = 4) but not in non-overexpressing mESCs (in EGFP mESCs-1 cell line, n = 4) (Fig 1D).

### Establishment of purified HCN4-overexpressing mESC-CMs

We generated 3 mESC lines in each group. In all mESCs, cardiac differentiation was performed well via EB formation with or without HCN4 overexpression. Cardiomyocytes were purified by changing culture media to glucose-free and lactic acid-supplemented media. Most of the collected cells spontaneously beat and expressed cardiac sarcomere proteins as assessed by immunostaining: non-overexpressing mESC-CMs, 91.6 ± 6.5% α-actinin positive and 90.2 ± 6.7% troponin I-positive; HCN4 overexpressing mESC-CMs, 86.2 ± 1.3% α-actinin positive and 88.8 ± 6.9% troponin I-positive. Proportions of α-actinin, troponin I, myosin light chain (MLC)-2v and MLC-2a-positive cells were not significantly different between the non-overexpression and HCN4 overexpression groups (Fig 2A).

**Fig 3:**
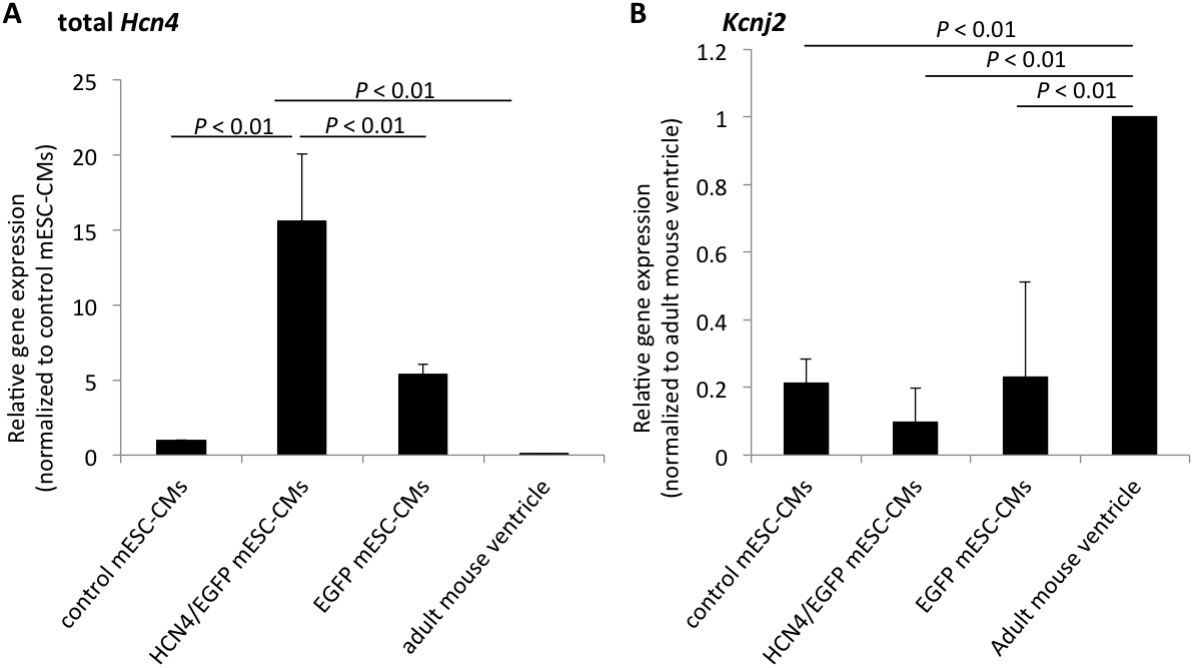
Quantitative PCR for total *Hcn4* and *Kcnj2* genes. A. Relative expression of total *Hcn4* gene. HCN4-overexpressing mESC-CMs expressed about 3-times higher mRNA levels of total *Hcn4* than did EGFP mESC-CMs. B. Relative expression of *Kcnj2* gene. In mESC-CMs, the expression level of *Kcnj2* was lower than that in an adult mouse ventricle.

RT-PCR showed that undifferentiated markers (*Oct4* and *Nanog*) had disappeared and that cardiac markers (*Nkx2*.*5* and *Tnnt2*) were positive in all 3 mESC-CM lines in each group (HCN4/EGFP or EGFP-stably-transfected mESC-CM group) (Fig 2B and S2 Fig). Expression of the *Cacna1h* gene, which encodes T type calcium channel and generates diastolic depolarization in the sinoatrial node, was also confirmed. Additionally, genes required for impulse conduction including *Connexin40*, *Connexin43*, and *Connexin45* genes, which encode connexins forming gap junctions, and the *Scn5a* gene, which encodes sodium channels, are expressed in the cells (Fig 2B and S2 Fig).

q-PCR showed that rabbit Hcn4 mRNA levels were not significantly different among the 3 HCN4/EGFP mESC-CM lines (S2B Fig). HCN4-overexpressing mESC-CMs (HCN4/EGFP mESC-CMs) expressed a 3-times higher level of *Hcn4* than did non-overexpressing mESC-CMs (EGFP mESC-CMs) (Fig 3A). Both HCN4-overexpressing and non-overexpressing mESC-CMs expressed lower levels of *Kcnj2*, which is involved in *I*_K1_ maintaining resting membrane potential, than did an adult mouse ventricle (Fig 3B).

**Fig 4:**
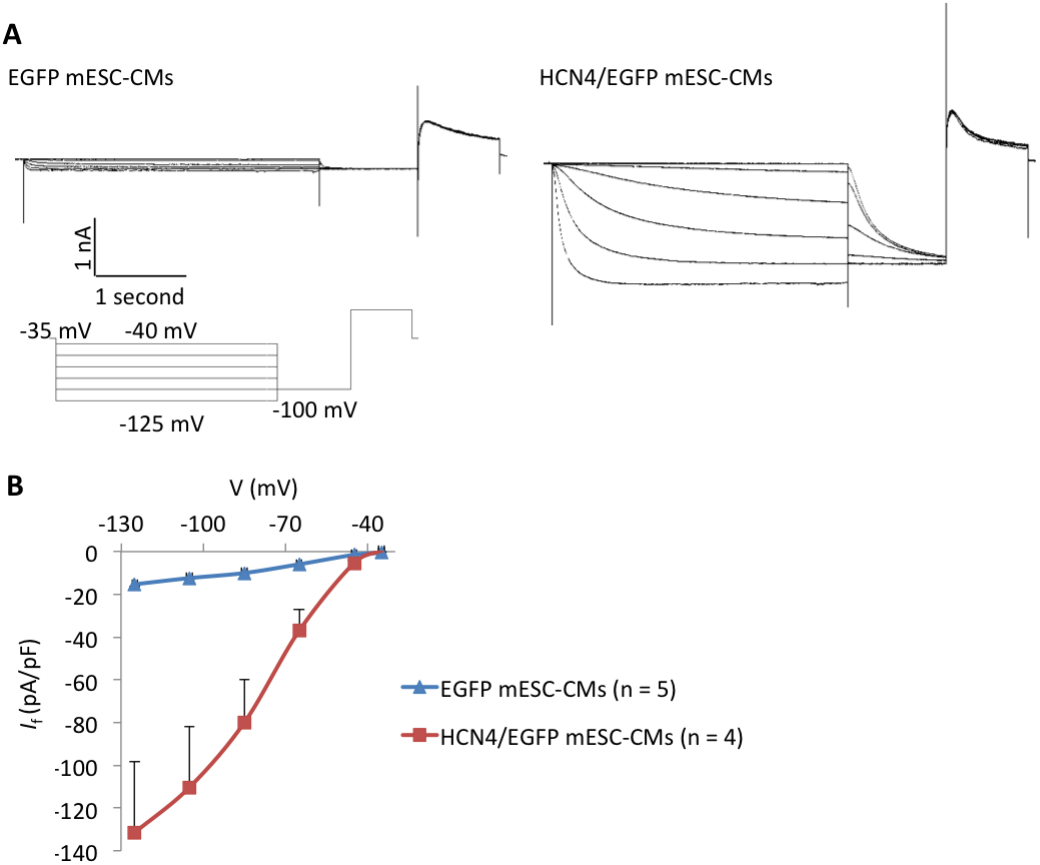
Measurement of *I*_f_ currents in mESC-CMs. A. Representative *I*_f_ currents in *EGFP*-(left) or *Hcn4/EGFP-*(right) overexpressing mESC-CMs. HCN4-overexpressing mESC-CMs showed a larger *I*_f_ current than did non-overexpressing cells. *I*_f_ currents through activated HCN channels were obtained during hyperpolarizing test pulses of 5 seconds between -45 and -125 mV in 20 mV increments from a holding potential of -35 mV. B. *I*_f_-V relationship curve in *EGFP*-(blue line) or *Hcn4/EGFP-*(red line) overexpressing mESC-CMs.

Furthermore, HCN4-overexpressing mESC-CMs (HCN4/EGFP mESC-CMs) showed a significantly larger *I*_f_ current than did non-overexpressing mESC-CMs (EGFP mESC-CMs) (Fig 4A and 4B).

### Rapid spontaneous beating in HCN4-overexpressing mESC-CMs

**Fig 5:**
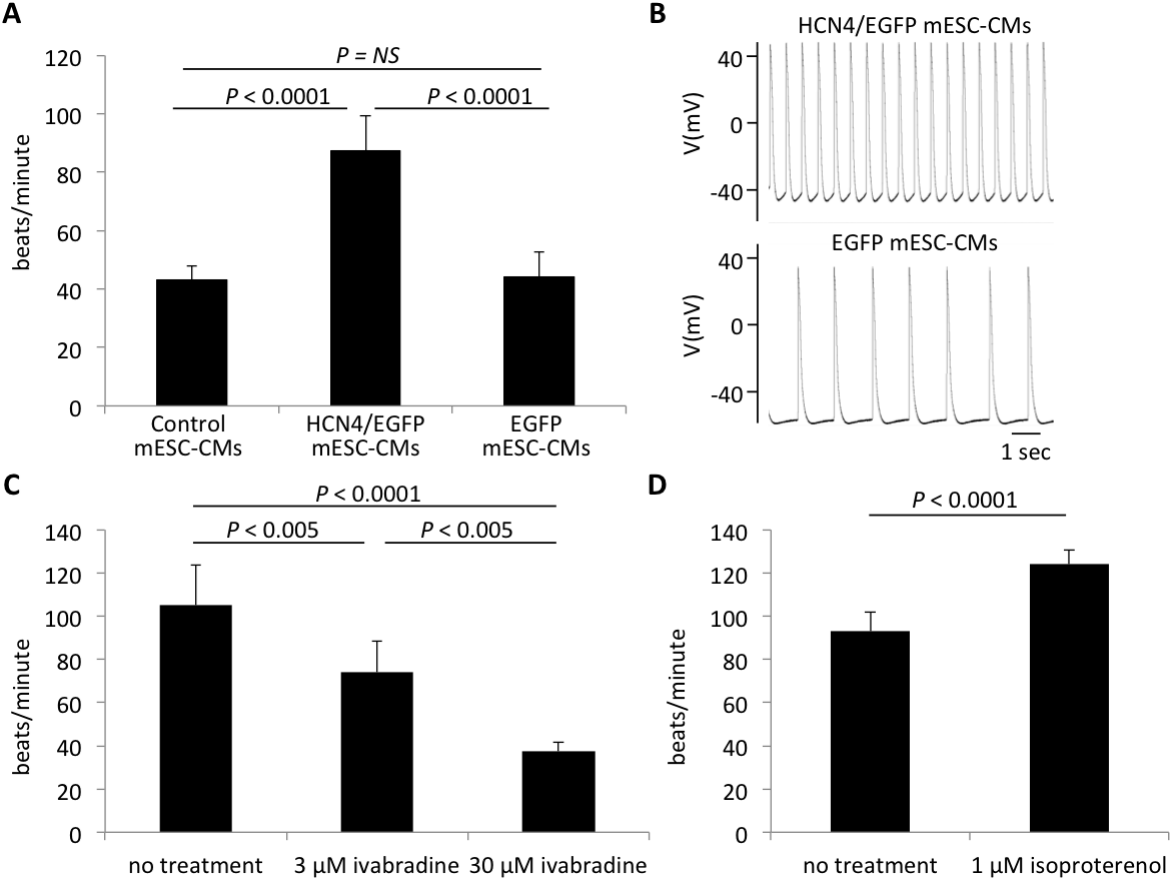
Spontaneous beating rates in mESC-CMs. A. Beating rates in control mESC-CMs, HCN4-overexpressing mESC-CMs (HCN4/EGFP mESC-CMs-2) and EGFP-stably-transfected mESC (EGFP mESC-CMs-2). HCN4-overexpressing mESC-CMs (HCN4/EGFP mESC-CMs-2) showed significantly more rapid beating than did non-overexpressing cells (Control and EGFP mEDC-CMs-2) (n = 8 per group). Data are expressed as mean ± SD. B. Representative action potentials in HCN4-overexpressing mESC-CMs (HCN4/EGFP mESC-CMs) (upper panel) and in non-overexpressing cells (EGFP mESC-CMs) (bottom panel). C and D. Beating rates of HCN4-overexpressing mESC-CMs decreased in response to ivabradine and increased in response to isoproterenol. (n = 6 in each group). Data are expressed as mean ± SD.

HCN4-overexpressing mESC-CMs showed significantly more rapid beating than did non-overexpressing mESC-CMs (Control mESC-CMs, 43.1 ± 4.8 beats/min; HCN4/EGFP mESC-CMs, 87.4 ± 11.9 beats/min; EGFP mESC-CMs, 44.3 ± 11.9 beats/min, n = 8 in each group, *P* < 0.0001) (Fig 5A and 5B).

The beating rate of HCN4-overexpressing mESC-CMs decreased in response to ivabradine, an agent with a selective and specific antagonistic effect on *I*_f_ currents, in a dose-dependent manner (no treatment, 105 ± 18.9 beats/min; 3 μM ivabradine, 74.0 ± 14.7 beats/min; 30 μM ivabradine, 37.5 ± 4.1 beats/min, n = 6 in each group, *P* < 0.005) (Fig 5C) and increased in response to isoproterenol, a beta-adrenergic receptor agonist (no treatment, 93.0 ± 8.9 beats/min; 1 μM isoproterenol, 124 ± 6.5 beats/min, n = 6 in each group, *P* < 0.0001) (Fig 5D).

### Rapid spontaneous beating in hiPSC-CMs synchronized with beating in HCN4-overexpressing mESC-CMs

We generated hiPSCs in which immunofluorescent staining showed nuclear accumulation of OCT4 and the expression pattern of cell surface markers: SSEA-1 negative, SSEA-4 positive, TRA-1-60 positive and TRA-1-81 positive (S4A Fig). Then we generated hiPSC-CMs that were positive for α-actinin and troponin I by immunofluorescent staining (S4B Fig).

**Fig 6:**
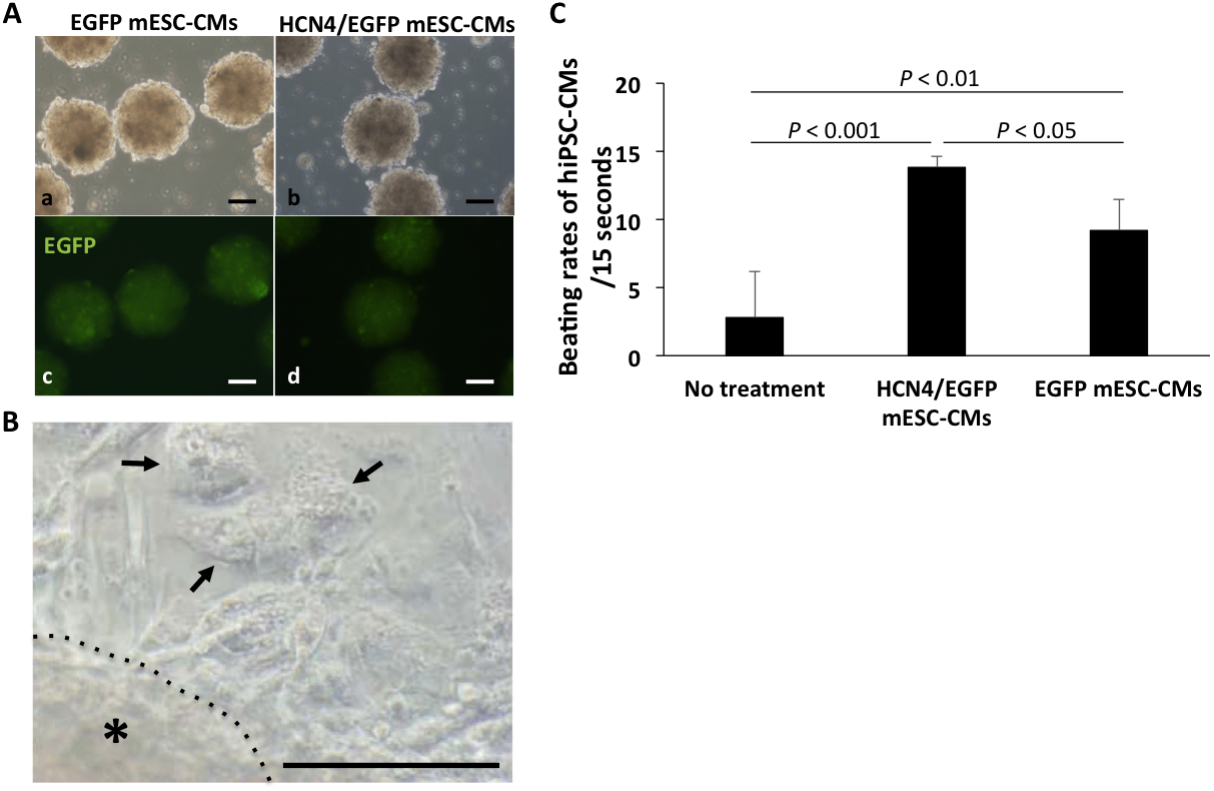
Spontaneous beating in hiPSC-CMs synchronized with beating in HCN4-overexpressing mESC-CMs. A. Cell aggregates composed of EGFP-stably-transfected mESCs (EGFP mESC-CMs) (a and c) and HCN4-overexpressing mESC-CMs (HCN4/EGFP mESC-CMs) (b and d) observed by phase contrast microscopy (a and b) and fluorescence microscopy (green: EGFP, c and d). Bar = 50 μm. B. hiPSC-CMs (arrows) co-cultured with aggregates of HCN4/EGFP mESC-CMs (*). Bar = 50 μm. C. Beating rates of hiPSC-CMs co-cultured with aggregates of HCN4/EGFP mESC-CMs and aggregates of EGFP mESC-CMs. Data are expressed as mean ± SD.

Co-culture of mESC-CMs with aggregates composed of mESC-CMs (Fig 6A) resulted in synchronized contraction of the cells. The beating rate of hiPSC-CMs co-cultured with aggregates of HCN4/EGFP mESC-CMs was significantly higher than that of non-treated hiPSC-CMs and that of hiPSC-CMs co-cultured with aggregates of EGFP mESC-CMs (not treated, 2.8 ± 3.3 beats/15 sec; co-cultured with aggregates of HCN4/EGFP mESC-CMs, 13.8 ± 0.8 beats/15 sec; co-cultured with aggregates of EGFP mESC-CMs, 9.2 ± 2.3 beats/15 sec, n = 5 in each group (not treated, n = 5; HCN4/EGFP mESC-CMs-1, n = 1; HCN4/EGFP-2, n = 2; HCN4/EGFP-3, n = 2 and EGFP mESC-CMs-1, n = 1; EGFP-2, n = 2; EGFP-3, n = 2)) (Fig 6B). These data showed that HCN4-overexpressing mESC-CMs could electronically couple and pace excitable cells *in vitro* and indicated that these cells could function as a biological pacemaker.

## Discussion

We established HCN4-overexpressing mESC-CMs as a candidate for a biological pacemaker. We generated the cells in order to achieve two prerequisites for forming spontaneous diastolic depolarization: large *I*_f_ currents and small *I*_K1_ currents. The cells have five specific abilities to become an appropriate biological pacemaker. (1) HCN4-overexpressing mESC-CMs expresses high levels of *Hcn4* and *Cacna1h* genes and a low level of the *Kcnj2* gene. (2) The cells show large *I*_f_ currents and rapid spontaneous beating, in other words, rapid impulse generation. (3) Additionally, genes required for impulse conduction including *Connexin40*, *Connexin43*, and *Connexin45* genes, which encode connexins forming gap junctions and the *Scn5a* gene, which encodes sodium channels, are expressed in the cells. (4) Furthermore, the beating of the cells responds to an *I*_f_ inhibitor and beta-adrenergic receptor agonist. (5) HCN4-overexpressing mESC-CMs have pacing ability in an *in vitro* co-culture system with other excitable cells.

Several investigators have reported overexpression of HCN channels as a strategy for generating a biological pacemaker. Injection of the *Hcn1-ΔΔΔ*, *Hcn2* or *Hcn4* gene [22–27] and transplantation of *Hcn2*- or *Hcn4*-overexpressing mesenchymal stem cells [28, 29] have been reported. Furthermore, *Tbx18* gene transfer in mature ventricular cardiomyocytes increases HCN4 channel expression and yields pacemaker activity [30]. We also generated HCN4-overexpressing mESC-CMs. The cells show large *I*_f_ currents. Enhancement of *I*_f_ currents flowing through HCN channels is a core strategy for generating a biological pacemaker. Mouse ESC-derived myocytes expressed *I*_f_ currents [31]. Since HCN4 expression gradually decreases in the late stage of cardiac differentiation [10, 32], there is concern about whether pacemaker function can be maintained in the long term. Therefore, we overexpressed HCN4 in ESC-CMs. Several investigators also isolated or induced sinoatrial node-like cells that express HCN4 from ESCs [33–35]. Although it is not clear which method is the most useful, our HCN4-overexpressing system is an effective and easy method to obtain a large *I*_f_ current and a large amount of cells.

Not only enhancement of *I*_f_ current through HCN channels but also attenuation of *I*_K1_ current and presence of other currents through T-type Ca^2+^ channels are required for diastolic depolarization [36, 37]. Additionally, subsequent propagation to the surrounding working myocardium through connexins and Na_v_1.5 channel is necessary as a pacemaker [38, 39]. Our HCN4-overexpressing mESC-CMs also expressed these channels.

Ivabradine is an *I*_f_ inhibitor and reduces the firing rate of pacemaker cells [40, 41], and it has already been applied in a clinical setting [42]. HCN4-overexpressing mESC-CMs showed responses to ivabradine. This drug could regulate tachyarrhythmia caused by hyperexcitability of these cells. In addition, HCN4-overexpressing mESC-CMs showed responses to isoproterenol, a β1- and β2-adrenoreceptor agonist, and might be useful to achieve heart rate response during exercise.

A sufficient number of cells is necessary to engraft well and capture the surrounding myocardium [28]. Since pluripotent stem cells have a strong self-renewal property, the use of pluripotent stem cell-derived cardiomyocytes is reasonable. Furthermore, hiPSCs are thought to be able to solve the likelihood of immune rejection [43, 44]. Thus, this HCN4-overexpressing method might be applicable to hiPSC-CMs.

Recently, Inada et al reported that the spatial heterogeneous nature of the sinus node is important for its normal functioning and that the presence of Na channel and connexin 43 in the periphery may be essential for the node to be able to drive the atrial muscle [38, 39]. Mouse ESC-CMs include a heterogeneous population: nodal-like, atrial myocyte-like and ventricular myocyte-like cells. Working myocyte-like cells from ESCs originally express Na channel and connexins. However, it is not obvious that spatial sorting would occur if ESC-CMs were injected *in vivo*. Further studies are needed to clarify this point.

Transplantation of human ESC-CMs into the ventricle of a complete atrioventricular block model animal has been reported [45]. Our experiment was only an *in vitro* experiment, and we need to evaluate the efficacy of these cells in bradycardia model animals.

## Conclusion

We generated HCN4-overexpresssing mESC-CMs showing rapid spontaneous beating, responses to drugs and improved pacing ability *in vitro*. The results indicated that these cells could be applied to a biological pacemaker.

## Supporting Information

S1 Fig Three mESC lines with or without HCN4 overexpression. A. Representative living *Hcn4/EGFP*-transfected mESCs observed by phase contrast microscopy (a to c) and fluorescence microscopy (d to f). *Hcn4/EGFP*-transfected mESCs were positive for EGFP (green) fluorescence (d to f). B. Representative living *EGFP*-transfected mESCs observed by phase contrast microscopy (a to c) and fluorescence microscopy (d to f). *EGFP*-transfected mESCs were positive for EGFP (green) fluorescence (d to f). C. Representative living control mESCs observed by phase contrast microscopy (a) and fluorescence microscopy (b). Control mESCs were negative for EGFP (b). Bar = 50 μm.
(TIF)

S2 Fig Expression of cardiac differentiation marker genes in mESC and mESC-CM lines. A. In all cell lines with or without HCN4 overexpression, RT-PCR showed increases in mRNA expression for cardiac markers *Nkx 2*.*5*, *Tnnt2*, *connexin*, *Scn5a*, *Cacna1h*, and mouse endogenous *Hcn4* (lanes 7 to 12). Rabbit exogenous *Hcn4* was expressed in HCN4/EGFP mESCs (lanes 4 to 6) and mESC-CMs (lane 10 to 12). B. Rabbit Hcn4 mRNA levels in 3 HCN4/EGFP mESC-CM lines assessed by q-PCR.
(TIF)

S3 Fig Expression of undifferentiated cell markers in HCN4/EGFP and EGFP mESCs. A. Immunofluorescent staining of OCT4 (a and b) and nuclear DNA staining by Hoecst (c and d) in HCN4/EGFP mESC-1 and 3 (immunofluorescent staining in HCN4/EGFP mESC-2 shown in Fig 1C). Bar = 50 μm. B. Immunofluorescent staining of OCT4 (a and b) and nuclear DNA staining by Hoecst (c and d) in EGFP mESC-2 and 3 (immunofluorescent staining in EGFP mESC-1 shown in Fig 1C). Bar = 50 μm. C. q-PCR showed that *Nanog* and *Oct4* mRNA levels were not significantly different in all mESC lines with or without HCN4 overexpression.
(TIF)

S4 Fig Generation of hiPSCs and hiPSC-CMs. A. Generated hiPSCs observed by phase contrast microscopy (a). Immunofluorescent staining showed nuclear accumulation of OCT4 (red) (b to d) and cell surface antigen expression (green) pattern of human pluripotent stem cells (e, SSEA-1 negative; f, SSEA-4 positive; g, TRA1-60 positive; and h, TRA-1-81 positive). Bar = 50 μm. B. Differentiated cardiomyocytes from hiPSCs were positive for α-actinin (green) (a) and troponin I (green) (d). Counter staining with f-actin (red) (band e) and merge (c and f). Bar = 50 μm.
(TIF)
