# Supplementary figures and images for: Enhancement of Spontaneous Activity by HCN4 Overexpression in Mouse Embryonic Stem Cell-Derived Cardiomyocytes - A Possible Biological Pacemaker
Source: PLoS One. 2015 Sep 18;10(9):e0138193. doi: 10.1371/journal.pone.0138193 (PMC4575154; doi:10.1371/journal.pone.0138193)

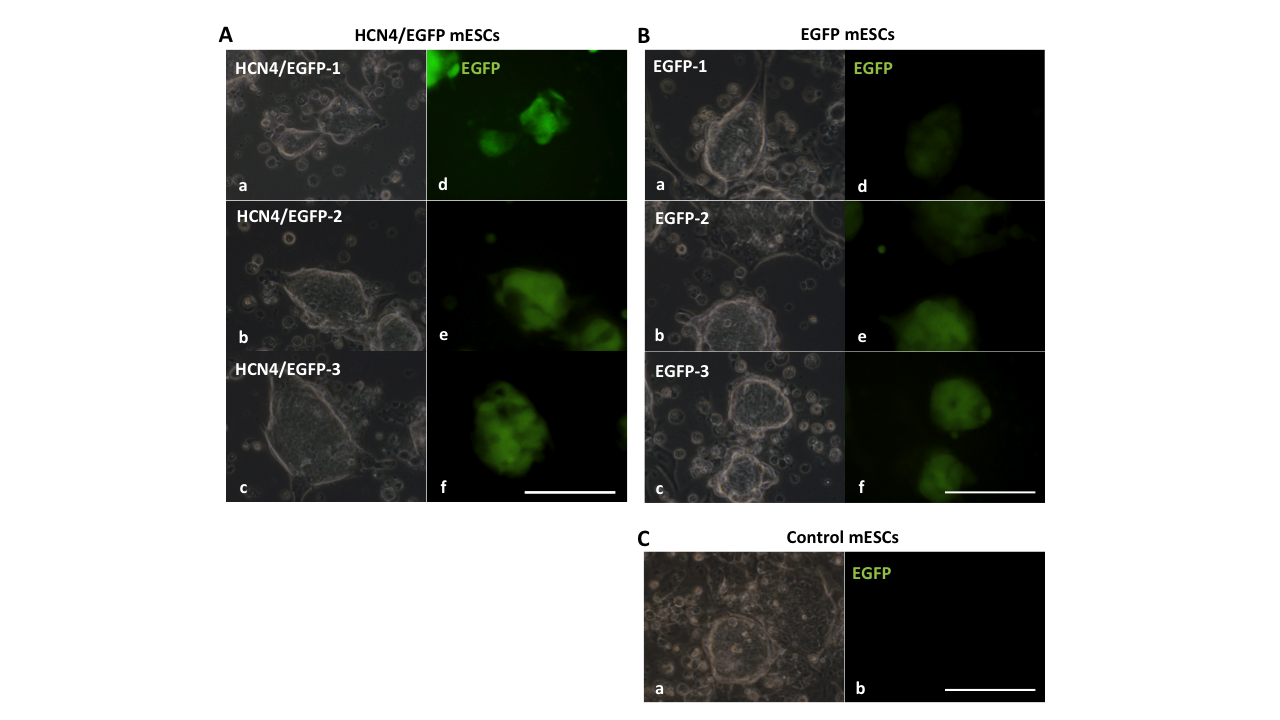

Supplement: S1 Fig — A. Representative living Hcn4/EGFP-transfected mESCs observed by phase contrast microscopy (a to c) and fluorescence microscopy (d to f). Hcn4/EGFP-transfected mESCs were positive for EGFP (green) fluorescence (d to f). B. Representative living EGFP-transfected mESCs observed by phase contrast microscopy (a to c) and fluorescence microscopy (d to f). EGFP-transfected mESCs were positive for EGFP (green) fluorescence (d to f). C. Representative living control mESCs observed by phase contrast microscopy (a) and fluorescence microscopy (b). Control mESCs were negative for EGFP (b). Bar = 50 μm. (TIF) [file pone.0138193.s001.tif]

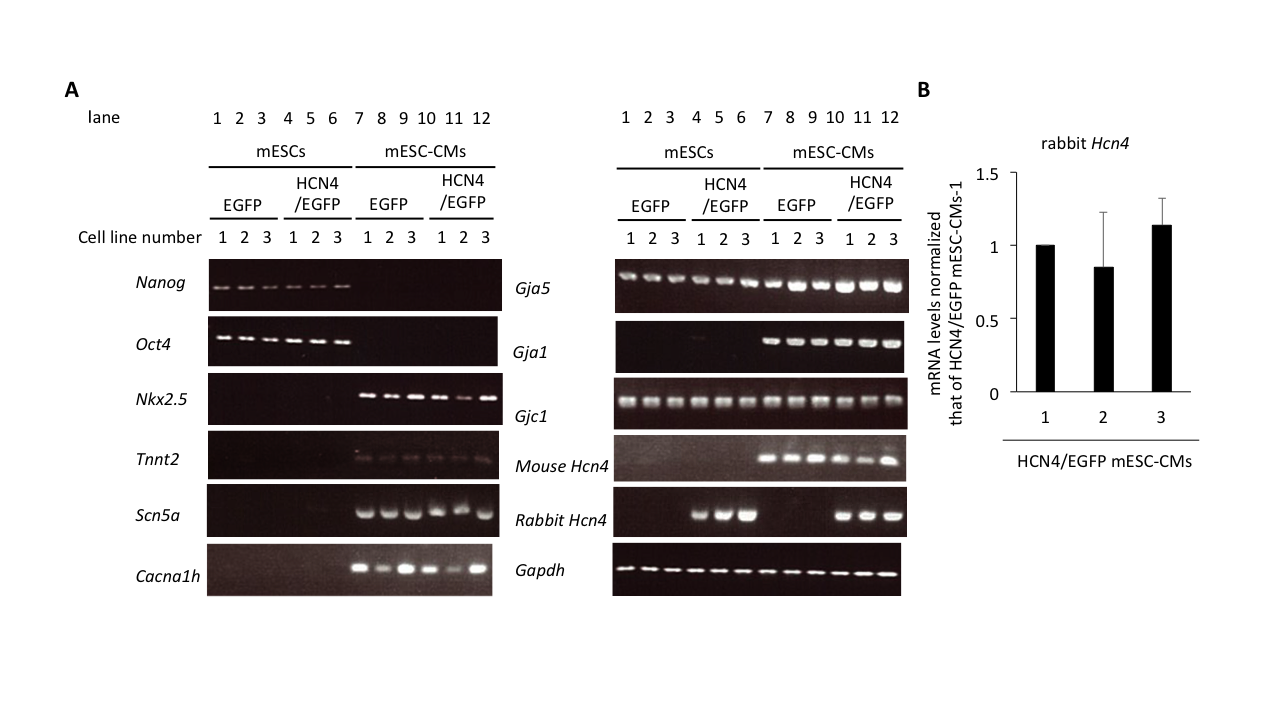

Supplement: S2 Fig — A. In all cell lines with or without HCN4 overexpression, RT-PCR showed increases in mRNA expression for cardiac markers Nkx 2.5, Tnnt2, connexin, Scn5a, Cacna1h, and mouse endogenous Hcn4 (lanes 7 to 12). Rabbit exogenous Hcn4 was expressed in HCN4/EGFP mESCs (lanes 4 to 6) and mESC-CMs (lane 10 to 12). B. Rabbit Hcn4 mRNA levels in 3 HCN4/EGFP mESC-CM lines assessed by q-PCR. (TIF) [file pone.0138193.s002.tif]

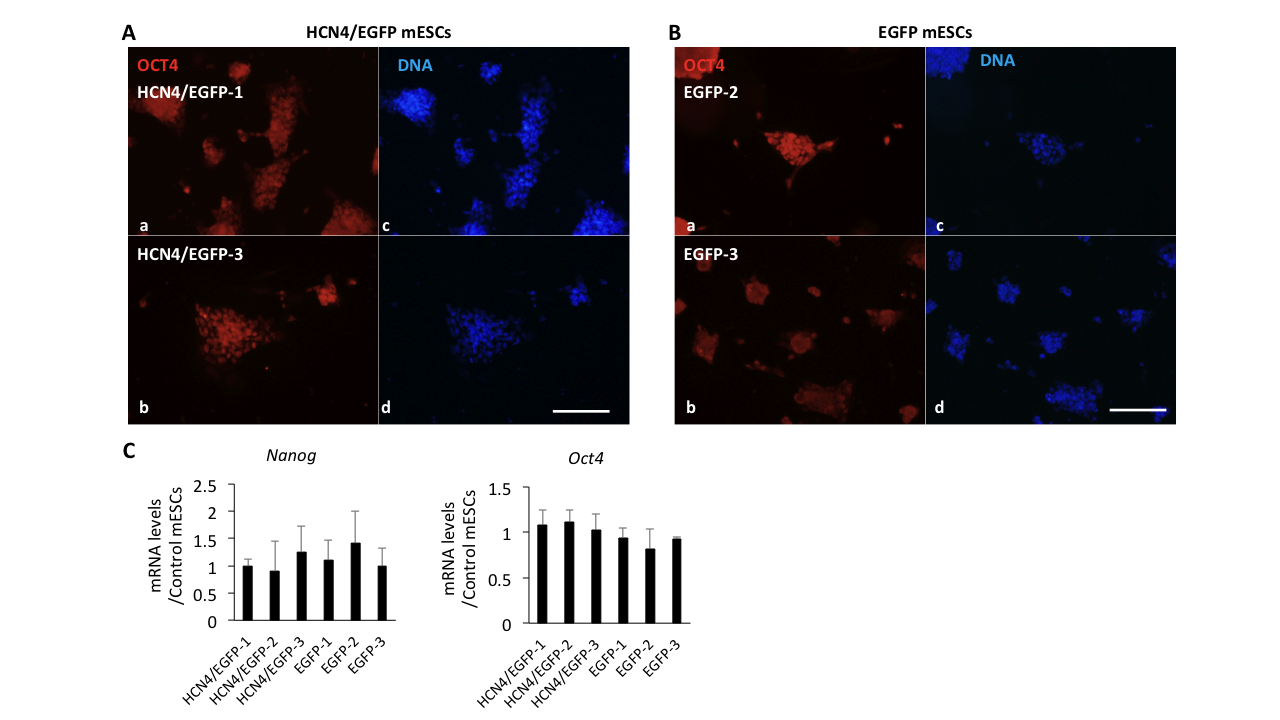

Supplement: S3 Fig — A. Immunofluorescent staining of OCT4 (a and b) and nuclear DNA staining by Hoecst (c and d) in HCN4/EGFP mESC-1 and 3 (immunofluorescent staining in HCN4/EGFP mESC-2 shown in Fig 1C). Bar = 50 μm. B. Immunofluorescent staining of OCT4 (a and b) and nuclear DNA staining by Hoecst (c and d) in EGFP mESC-2 and 3 (immunofluorescent staining in EGFP mESC-1 shown in Fig 1C). Bar = 50 μm. C. q-PCR showed that Nanog and Oct4 mRNA levels were not significantly different in all mESC lines with or without HCN4 overexpression. (TIF) [file pone.0138193.s003.tif]

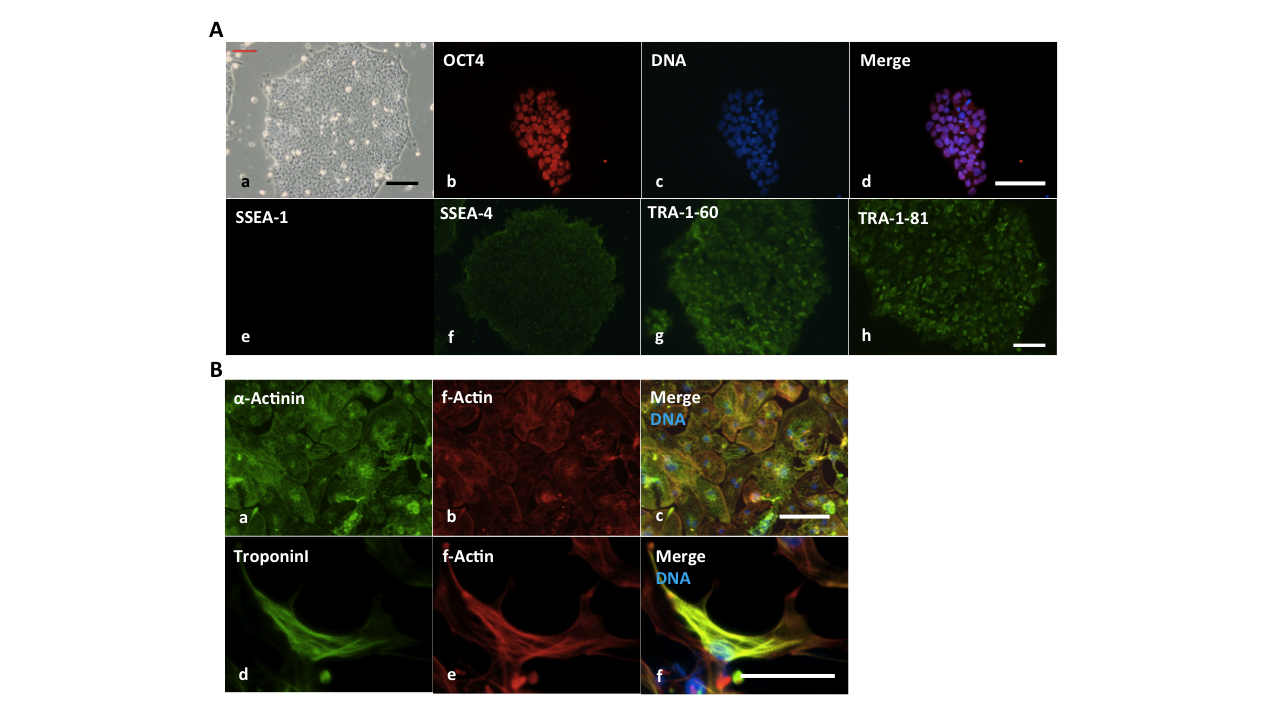

Supplement: S4 Fig — A. Generated hiPSCs observed by phase contrast microscopy (a). Immunofluorescent staining showed nuclear accumulation of OCT4 (red) (b to d) and cell surface antigen expression (green) pattern of human pluripotent stem cells (e, SSEA-1 negative; f, SSEA-4 positive; g, TRA1-60 positive; and h, TRA-1-81 positive). Bar = 50 μm. B. Differentiated cardiomyocytes from hiPSCs were positive for α-actinin (green) (a) and troponin I (green) (d). Counter staining with f-actin (red) (band e) and merge (c and f). Bar = 50 μm. (TIF) [file pone.0138193.s004.tif]
